# High-Performance Sn^2+^-Doped CuFe_2_O_4_-Based Resistance Gas Sensor for the Detection of the Sarin Simulant DMMP

**DOI:** 10.3390/s25103042

**Published:** 2025-05-12

**Authors:** Junchao Yang, Liu Yang, Ting Liang, Ling Zhang, Jianan Wei, Shuya Cao, Qibin Huang

**Affiliations:** 1 State Key Laboratory of Chemistry for NBC Hazards Protection, Beijing 102205, China; yangjunchao1990@163.com (J.Y.); yangliujinjin@sina.com (L.Y.); zhanglingscdx@163.com (L.Z.); weijianan216@163.com (J.W.); 2 Institute of NBC Defense, Beijing 102205, China; 18511090259@163.com

**Keywords:** CuFe_2_O_4_, Sn^2+^ doping, chemical toxins, DMMP, gas sensor

## Abstract

**Highlights:**

**What are the main findings?**

- A 2 mol% Sn^2+^-doped CuFe_2_O_4_ sensor was produced for DMMP detection.
- The sensor demonstrated suitable sensing performance and selectivity toward DMMP.

**What is the implication of the main finding?**

- The superior response–recovery characteristics and enhanced moisture resistance compared to the undoped sensor are relevant in gas-sensing applications.
- Sarin poses a serious threat to human health, necessitating the development of an appropriate sensing technology.

**Abstract:**

Sarin is an extremely toxic and fast-acting chemical warfare nerve agent that poses a serious threat to human health, necessitating the development of appropriate sensing technologies. Dimethyl methylphosphonate (DMMP), which has a chemical structure similar to that of sarin but is non-toxic, is often used as a simulation agent in related research. Among promising gas-sensing materials, CuFe_2_O_4_ exhibits suitable thermal stability. It is easily produced and has low toxicity. Its performance can be enhanced using heterogeneous ion doping to increase the number of surface defects and content of adsorbed oxygen. Therefore, a solvothermal method was adopted in this study to prepare CuFe_2_O_4_ hollow microspheres that were subsequently doped with different ratios of Sn^4+^ or Sn^2+^. Detailed characterizations of the obtained materials were conducted, and the corresponding CuFe_2_O_4_-based gas sensors were fabricated. Their gas-sensing performance against DMMP was studied to analyze and discuss the gas-sensing and sensitization mechanisms associated with Sn^4+^ and Sn^2+^ doping. The CuFe_2_O_4_-based sensor doped with 2 mol% Sn^2+^ exhibited excellent gas-sensing performance in response to a 1 ppm concentration of DMMP, with response and recovery times of 12 and 63 s, respectively. Notably, its response to 1 ppm DMMP (16.27) was 3.3-fold higher than that to 1 ppm 2-CEES (4.98). The doped CuFe_2_O_4_ sensor exhibited superior response–recovery characteristics and enhanced moisture resistance compared to the undoped sensor.

## 1. Introduction

Sarin is a representative and highly toxic nerve agent that is widely used in chemical warfare. Considering its extremely low immediately dangerous to life or health (IDLH) value of 0.05 ppm [1], the rapid and accurate detection of sarin is critical [2,3]. Dimethyl methylphosphonate (DMMP) is commonly used as a simulation agent [4,5,6] in sarin-related research under laboratory conditions because it has a similar chemical structure to sarin but is non-toxic. At present, the primary detection methods for such chemical agents and their mimetics include laser spectroscopy [7], infrared spectroscopy [8], Raman spectroscopy [9], ion mobility spectroscopy [10], mass spectrometry [11], surface acoustic wave sensors [12], and metal-oxide semiconductor gas sensors [13,14,15]. Among these, metal-oxide semiconductor sensors have been successfully deployed to detect low concentrations of harmful gases owing to their simple structures, low cost nature, and facile miniaturization [16,17,18].

Among promising gas-sensing materials, spinel-structured CuFe_2_O_4_ exhibits suitable thermal stability, facile producibility, and low toxicity [19,20,21]. The performance of such materials can be enhanced by heterogeneous ion doping, which increases the number of surface defects, as well as the adsorbed oxygen ratio, while regulating the carrier concentration [22,23,24]. Among potential dopants for the CuFe_2_O_4_ lattice, Sn^4+^ (0.71 Å) and Cu^2+^ (0.72 Å) possess similar ionic radii, whereas the radius of Sn^2+^ (0.93 Å) is significantly larger than that of either Cu^2+^ (0.72 Å) or Fe^3+^ (0.64 Å). Consequently, Sn^4+^ can replace Cu^2+^ for substitutional doping, whereas Sn^2+^ can be doped into the gaps of the CuFe_2_O_4_ lattice to realize interstitial doping.

Therefore, the objective of this study was to prepare graded CuFe_2_O_4_ hollow microspheres using a solvothermal method and subsequently dope this material with different concentrations of Sn^4+^ or Sn^2+^ to prepare gas-sensing materials. Detailed characterization tests of these materials were conducted, the corresponding gas sensors were fabricated, and their DMMP-sensing performance was evaluated to analyze the gas-sensing and sensitization mechanisms associated with Sn^4+^ and Sn^2+^ doping.

## 2. Materials and Methods

### 2.1. Materials and Reagents

The reagents employed in this study, including anhydrous copper nitrate (Cu(NO_3_)_2_, AR, ≥99%), iron nitrate nonahydrate (Fe(NO_3_)_3_·9H_2_O, AR, ≥98.5%), stannous chloride pentahydrate (SnCl_4_·5H_2_O, AR, ≥99%), anhydrous stannous chloride (SnCl_2_, AR, ≥99%), urea (CO(NH_2_)_2_, AR, ≥99%), glycerol (C_3_H_8_O_3_, AR), isopropanol (C_3_H_8_O, AR), and ethanol (C_2_H_5_OH, AR), were purchased from China National Pharmaceutical Chemical Reagent Co., Ltd. (Beijing, China). The chemical agent mimetics DMMP (C_3_H_9_O_3_P, AR, ≥98.5%) and 2-chloroethyl ethyl sulfide (2-CEES, C_4_H_9_ClS, AR, ≥98.5%) were obtained from Aladdin Reagent Co., Ltd. (Shanghai, China). The chemical agents sarin (GB, ≥98%) and mustard gas (HD, ≥98%) were procured from the Institute of Chemical Defense at the Academy of Military Sciences. The high-purity reagents used for testing, including methanol, ethanol, acetone, dichloromethane, trichloromethane, toluene, and n-hexane (>99%), were purchased from Beijing Hepu North Branch Gas Industry Co., Ltd. (Beijing, China). All samples were used as received without further purification.

### 2.2. Material Synthesis and Preparation

#### 2.2.1. Synthesis and Preparation of CuFe_2_O_4_ Doped with Different Ratios of Sn^4+^

SnCl_4_·5H_2_O (0.350 g) was placed in a 50 mL volumetric flask and isopropanol was added to make up the volume. After being shaken well, this flask was set aside until required for later use. Next, Cu(NO_3_)_2_ (0.094 g, 0.5 mmol), Fe(NO_3_)_3_·9H_2_O (0.404 g, 1.0 mmol), and urea (0.060 g, 1.0 mmol) were added to a mixed solvent of glycerol (4 mL) and isopropanol (16 mL). Subsequently, the desired volume of the prepared SnCl_4_·5H_2_O solution (0, 250, 375, 500, 750, or 100 µL) was added, and the mixture was stirred magnetically for 30 min. The resulting solution was transferred to a 50 mL reaction vessel for hydrothermal reaction at 180 °C for 24 h. The obtained solution was subjected to alternative centrifugation at 9000 rpm six times using deionized water and ethanol. The centrifuged sample was subsequently dried for 12 h at 80 °C in a vacuum drying oven. Finally, the dried sample was placed in a muffle furnace, heated to 450 °C at a rate of 2 °C/min under an air atmosphere, and calcined at this target temperature for 2 h to obtain the undoped and 1, 1.5, 2, 3, and 4 mol% Sn^4+^-doped CuFe_2_O_4_ materials.

#### 2.2.2. Synthesis and Preparation of CuFe_2_O_4_ Doped with Different Ratios of Sn^2+^

SnCl_2_ (0.190 g) was placed in a 50 mL volumetric flask and isopropanol was added to make up the volume. After being shaken well, this flask was set aside until required for later use. Next, Cu(NO_3_)_2_ (0.094 g, 0.5 mmol), Fe(NO_3_)_3_·9H_2_O (0.404 g, 1.0 mmol), and urea (0.060 g, 1.0 mmol) were added to a mixed solvent of glycerol (4 mL) and isopropanol (16 mL). Subsequently, the desired volume of the prepared SnCl_2_ solution (0, 250, 375, 500, 750, or 100 µL) was added, and the mixture was stirred magnetically for 30 min. The resulting solution was transferred to a 50 mL reaction vessel for hydrothermal reaction at 180 °C for 24 h. The obtained solution was subjected to alternative centrifugation at 9000 rpm six times using deionized water and ethanol. The centrifuged sample was subsequently dried at 80 °C for 12 h in a vacuum drying oven. Finally, the dried sample was placed in a muffle furnace, heated to 450 °C at a rate of 2 °C/min under an air atmosphere, and calcined at this target temperature for 2 h to obtain the undoped and 1, 1.5, 2, 3, and 4 mol% Sn^2+^-doped CuFe_2_O_4_ materials.

### 2.3. Characterization of the Prepared Materials

The structural morphology of the samples was characterized using scanning electron microscopy (SEM, ZEISS Gemini 300, Carl Zeiss, Jena, Germany) and transmission electron microscopy (TEM, JEM-F200, JEOL, Tokyo, Japan). The crystal phases of the samples were analyzed using X-ray diffractometry (XRD, Panaco Empyrean Sharp Shadow, Panalytical, Almelo, The Netherlands) with a Cu target and Kα radiation (λ = 0.154056 nm). The elemental composition of the samples was analyzed using energy dispersive X-ray spectrometry (EDS, JED-2300T, JEOL, Tokyo, Japan). The chemical composition of the samples was analyzed using X-ray photoelectron spectrometry (XPS, Thermo Scientific K-Alpha, Thermo Fisher Scientific, Waltham, MA, USA) with a working voltage of 12 V and a filament current of 6 mA; the electron binding energy was corrected using the C 1s peak of adventitious carbon (284.8 eV).

### 2.4. Manufacturing and Testing of Gas Sensors

**Figure 1:**
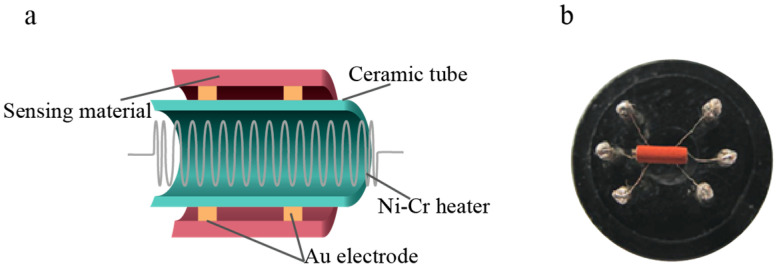
Schematic of the gas sensor device. (**a**) Cross-sectional structure of the sensor. (**b**) Physical image of the sensor [13].

Gas sensors were produced by adding the desired CuFe_2_O_4_ material to an appropriate quantity of deionized water to form a uniform slurry. A fine brush was used to evenly coat a thin film of this slurry onto an alumina ceramic tube, which was equipped with Au electrodes at both ends and a Pt wire serving as a lead to form the test electrode. After drying at room temperature, the tube was sintered at 300 °C for 2 h. Finally, a Ni-Cr alloy heating wire was placed inside the ceramic tube and welded to a six-legged base, as shown in Figure 1a,b.

**Figure 2:**
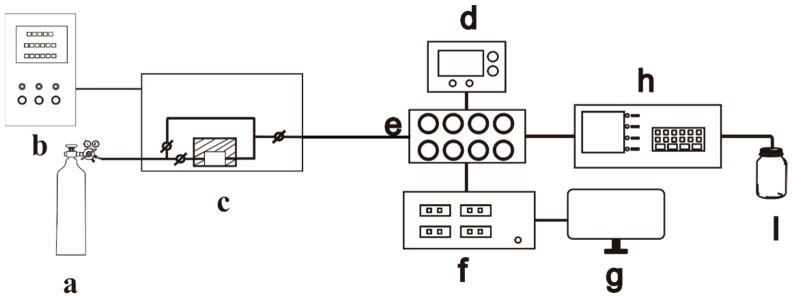
Dynamic testing platform: (**a**) gas cylinder, (**b**) system controller, (**c**) dynamic preparation system, (**d**) stabilized DC power supply, (**e**) eight-channel testing chamber, (**f**) test source table, (**g**) workstation, (**h**) concentration monitor, and (**i**) waste gas cylinder [13].

Each gas sensor was first aged on an aging table for >72 h, then placed in an eight-channel testing chamber for evaluation (Figure 2e). The desired chemical or simulation agent was prepared in the component represented in Figure 2c, where the concentration of the target gas was controlled by adjusting the temperature and gas flow rate in the generating pool using the controller (Figure 2b). The voltage source (Figure 2d) was used to control the working temperature of the gas component by adjusting the temperature of the heating wire. The monitoring instrument (Figure 2h) provided real-time concentration information. The workstation (Figure 2g) was employed to obtain real-time resistance data from the test source table (Figure 2f) for subsequent data processing. The response of the sensor was defined as *S* = *R*_a_/*R*_g_, where *R*_a_ and *R*_g_ are the resistances of the device in air and in a stable state under the test gas atmosphere, respectively. The response time (*t*_res_) and recovery time (*t*_rec_) of the device were defined as the times required for the gas-sensing element to achieve a total resistance change of 90% during the adsorption and desorption processes, respectively.

## 3. Results and Discussion

### 3.1. Structure and Morphology

**Figure 3:**
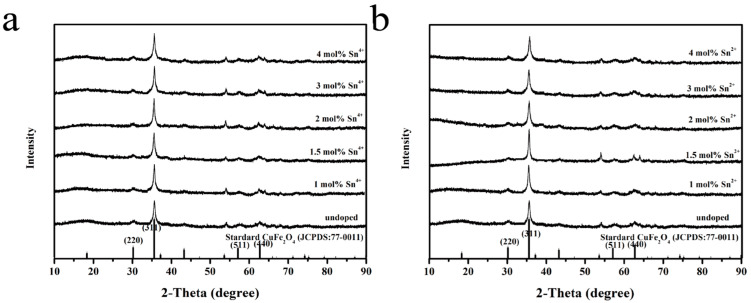
XRD patterns of CuFe_2_O_4_ doped with different ratios of (**a**) Sn^4+^ and (**b**) Sn^2+^.

The XRD patterns of the CuFe_2_O_4_ samples doped with different ratios of Sn^4+^ and Sn^2+^ are shown in Figure 3a and Figure 3b, respectively. Diffraction peaks were observed for the prepared samples at 2θ = 30.17°, 35.54°, 57.13°, and 62.74°, corresponding to the (220), (311), (511), and (440) crystal planes, respectively, of the CuFe_2_O_4_ standard diffraction pattern Standard CuFe_2_O_4_ (JCPDS: 77-0011).(Xueli Yang, Jilin University, China, 2019). Notably, no peaks corresponding to SnO_2_ impurities or other crystal phases were observed in any of the doped materials, confirming successful doping. In addition, the diffraction peaks of the doped samples were found to broaden (i.e., exhibit larger *β* values) with increasing doping levels. According to the Scherrer formula (*D* = 0.89*λ*/*β*cos*θ*, where *D* is the grain size, *λ* is the wavelength of the incident X-ray, *β* is the half width of the diffraction peak, and *θ* is the Bragg diffraction angle), this result indicates that the sample grain size gradually decreased with increasing ion doping concentration, likely owing to inhibited CuFe_2_O_4_ lattice growth upon Sn^4+^ and Sn^2+^ doping.

Subsequently, the CuFe_2_O_4_ samples doped with Sn^4+^ and Sn^2+^ were subjected to EDS; their corresponding elemental compositions are presented in Table 1 and Table 2, respectively. Table 1 indicates that the atomic fraction of Sn increased from 0% to 0.33% as the Sn^4+^ doping level increased from 0 to 4 mol%. Similarly, Table 2 indicates that the atomic fraction of Sn increased from 0% to 0.34% as the Sn^2+^ doping level increased from 0 to 4 mol%. These atomic fractions were close to the theoretical values in terms of the molar percentages of Sn^2+^ and CuFe_2_O_4_, thereby confirming the application of a feasible doping strategy.

**Figure 4:**
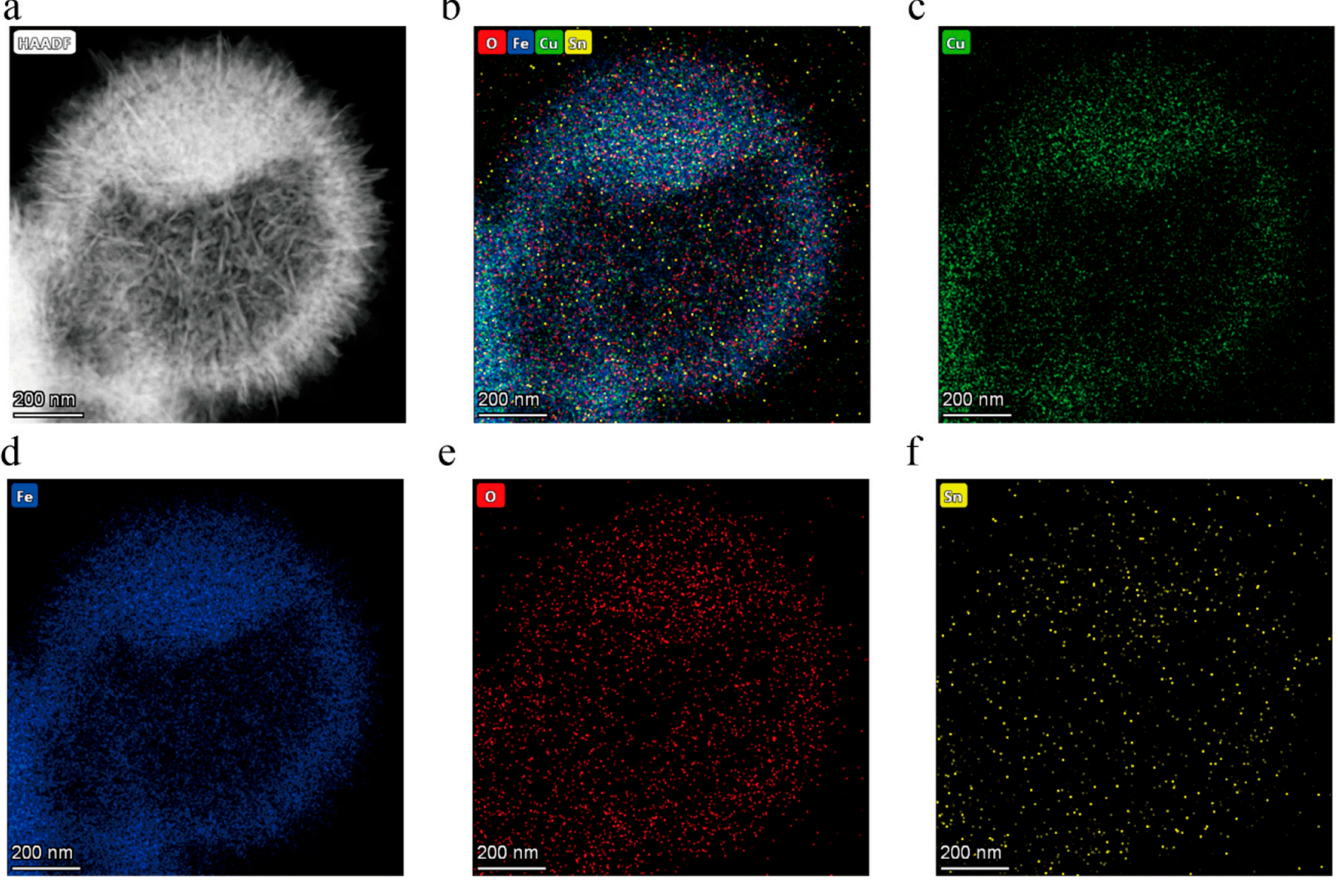
EDS mapping images recorded for the CuFe_2_O_4_ sample doped with 2 mol% Sn^4+^ (**a**) Sample topography, (**b**) Sample composition, (**c**) Cu, (**d**) Fe, (**e**) O, and (**f**) 2 mol% Sn^4+^.

**Figure 5:**
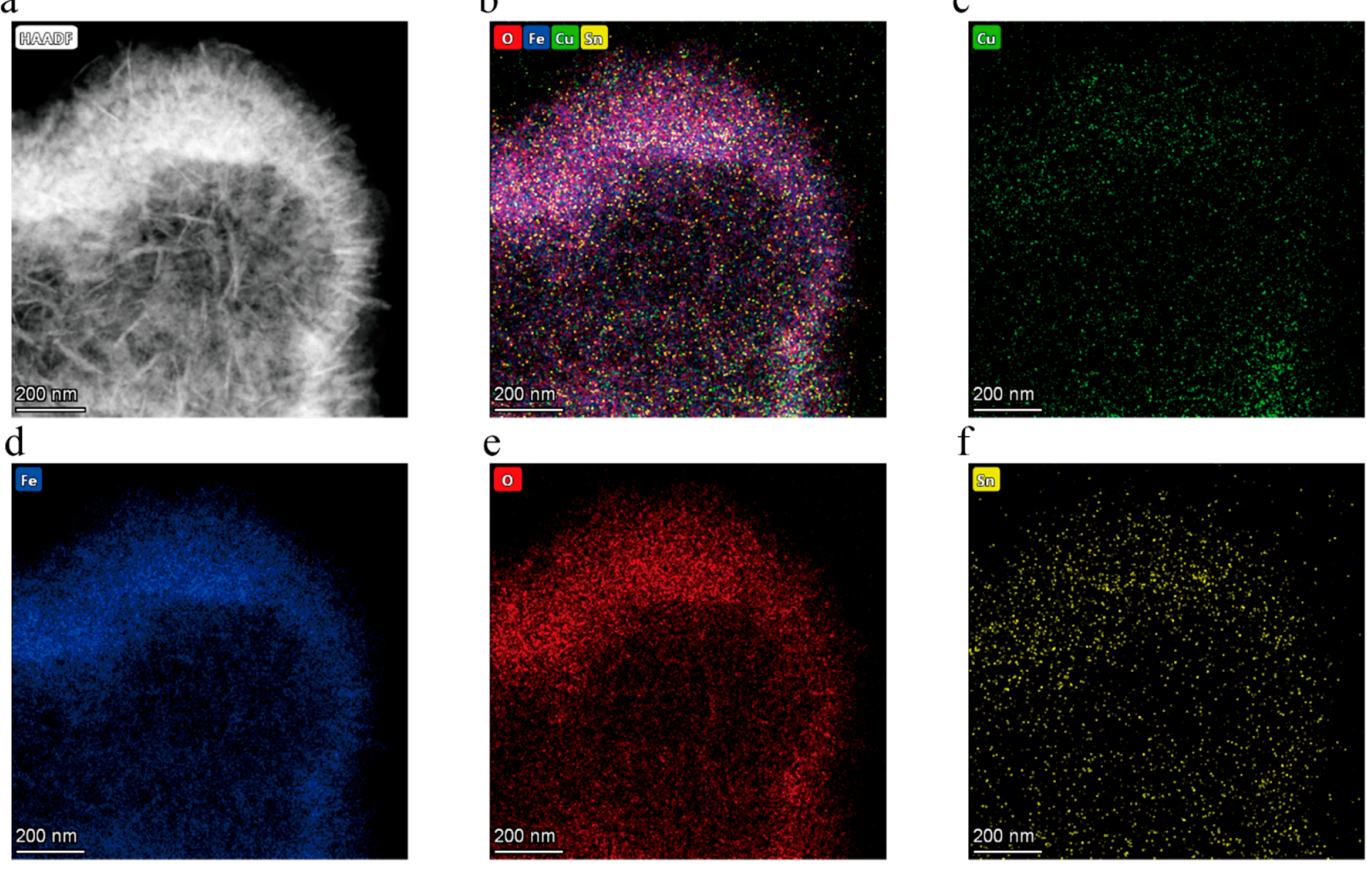
EDS mapping images recorded for the CuFe_2_O_4_ sample doped with 2 mol% Sn^2+^ (**a**) Sample topography, (**b**) Sample composition, (**c**) Cu, (**d**) Fe, (**e**) O, and (**f**) 2 mol% Sn^2+^.

The EDS mapping images of the CuFe_2_O_4_ species doped with 2 mol% Sn^4+^ and Sn^2+^ are shown in Figure 4 and Figure 5, respectively. These images indicate that Cu, Fe, O, and Sn were uniformly distributed throughout the CuFe_2_O_4_ hollow microsphere, confirming that uniform doping of Sn^4+^ and Sn^2+^ was achieved. As expected, the more intense signals observed for Cu, Fe, and O correlated with the significantly higher levels of these elements in the prepared materials compared to the levels of Sn^4+^ and Sn^2+^.

**Figure 6:**
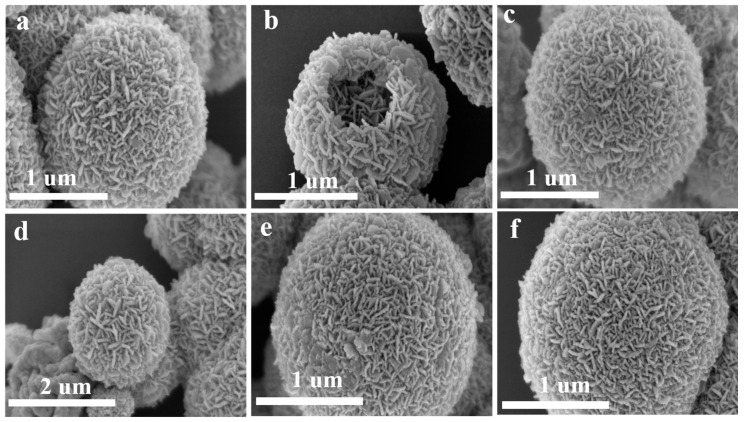
SEM images of the CuFe_2_O_4_ material doped with different Sn^4+^ ratios: (**a**) undoped, (**b**) 1 mol% Sn^4+^, (**c**) 1.5 mol% Sn^4+^, (**d**) 2 mol% Sn^4+^, (**e**) 3 mol% Sn^4+^, and (**f**) 4 mol% Sn^4+^.

**Figure 7:**
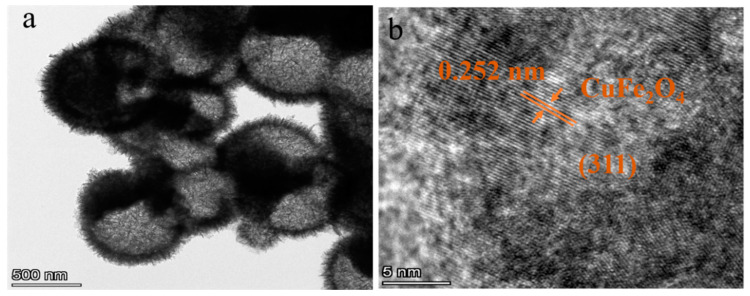
(**a**) TEM image and (**b**) HRTEM image of the 2 mol% Sn^4+^-doped CuFe_2_O_4_ sample.

Figure 6 shows the SEM images obtained from the six Sn^4+^-doped CuFe_2_O_4_ samples. All samples clearly exhibit hollow microsphere morphologies with rough surfaces and diameters of ~1.2 µm. In addition, the hierarchical structures of these microspheres can be observed to comprise assemblies of numerous two-dimensional (2D) porous nanosheets. Clear gaps exist between the nanosheets on the microsphere surface, indicating a loose and porous structure. Consequently, these materials can be expected to effectively adsorb gas molecules and promote their diffusion, facilitating gas-sensing performance. Notably, the microsphere size, hollow structure, and surface morphology did not change significantly as the level of Sn^4+^ doping increased. Furthermore, a sharp contrast between dark edges and relatively bright gaps can be observed in the TEM image of the CuFe_2_O_4_ sample doped with 2 mol% Sn^4+^ (Figure 7a), further confirming the hollow microsphere morphology. Finally, the high-resolution (HR) TEM image in Figure 7b exhibits clear lattice fringes spaced at 0.252 nm; this measurement corresponds to the (311) interplanar spacing of CuFe_2_O_4_, which is consistent with the XRD results.

**Figure 8:**
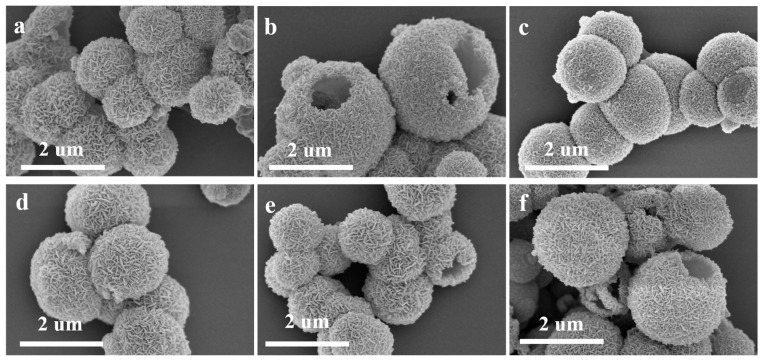
SEM images of the CuFe_2_O_4_ materials doped with different Sn^2+^ ratios: (**a**) undoped, (**b**) 1 mol% Sn^2+^, (**c**) 1.5 mol% Sn^2+^, (**d**) 2 mol% Sn^2+^, (**e**) 3 mol% Sn^2+^, and (**f**) 4 mol% Sn^2+^.

The SEM images of the six Sn^2+^-doped CuFe_2_O_4_ samples in Figure 8 indicate that all samples comprised hollow microspheres with rough surfaces and diameters of ~1.2 µm. Similar hierarchical structures composed of numerous assembled 2D porous nanosheets are also evident in these samples, as are clear gaps between the surface nanosheets, which can be expected to promote gas adsorption and diffusion. As was the case for the Sn^4+^-doped samples, where the microsphere size, hollow structure, and surface morphology did not change significantly with the Sn^2+^ content.

**Figure 9:**
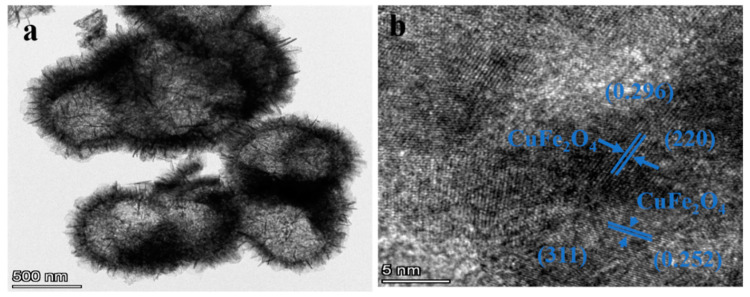
(**a**) TEM image and (**b**) HRTEM image of the 2 mol% Sn^2+^-doped CuFe_2_O_4_ sample.

Figure 9a shows a TEM image of the CuFe_2_O_4_ sample doped with 2 mol% Sn^2+^, confirming the presence of hollow microsphere structures with rough surfaces. The corresponding HRTEM image (Figure 9b) depicts clear lattice fringes spaced at 0.252 and 0.296 nm, corresponding to the (311) and (220) interplanar spacings, respectively, of the CuFe_2_O_4_ sample, which was consistent with the XRD results.

**Figure 10:**
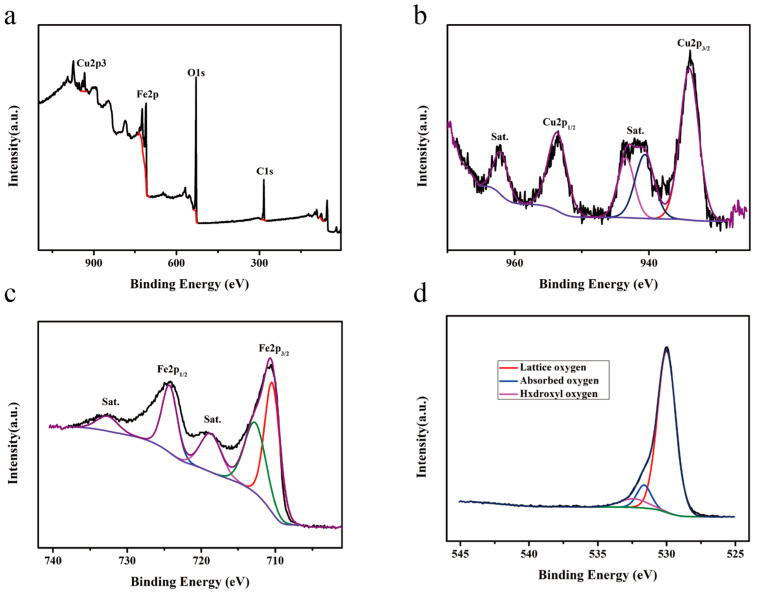
XPS spectra of the undoped CuFe_2_O_4_ hollow microsphere sample: (**a**) full survey spectrum, (**b**) Cu 2p spectrum, (**c**) Fe 2p spectrum, and (**d**) O 1s spectrum.

The XPS spectra of the undoped CuFe_2_O_4_ hollow microsphere samples are shown in Figure 10. All sample peaks were corrected using the C 1s peak with a binding energy of 284.8 eV. Figure 10a shows the full spectrum of the CuFe_2_O_4_ sample, in which characteristic Cu 2p, Fe 2p, O 1s, and C 1s peaks can be observed, further confirming the presence of Cu, Fe, O, and C, the last of which originated from the activated carbon introduced by the XPS instrument [25]. In the deconvoluted Cu 2p spectrum (Figure 10b), the peaks at 953.63 and 934.02 eV correspond to the Cu 2p_1/2_ and Cu 2p_3/2_ orbitals, respectively. The peaks at 962.06, 943.40, and 940.63 eV represent vibrational satellite peaks that are consistent with previous literature and indicate that Cu existed in the +2 valence state in the CuFe_2_O_4_ sample [26]. In the deconvoluted Fe 2p spectrum (Figure 10c), the peaks at binding energies of 712.68 and 710.41 eV can be attributed to Fe 2p_3/2_, that at 724.28 eV is assigned to Fe 2p_1/2_, and those at 732.65 and 718.74 eV correspond to the vibrational satellite peaks of Fe 2p; these results indicate that Fe was present in the +3 valence state [27]. In the deconvoluted O 1s spectrum (Figure 10d), the peaks located at 532.84, 531.69, and 530.02 eV correspond to hydroxyl oxygen, surface-adsorbed oxygen, and lattice oxygen species, respectively, [28] with respective proportions of 9.43%, 11.28%, and 79.29%, as determined by measuring the corresponding XPS peak areas. These XPS results were consistent with the corresponding XRD results presented in Figure 3, further confirming the successful preparation of hollow CuFe_2_O_4_ microspheres.

**Figure 11:**
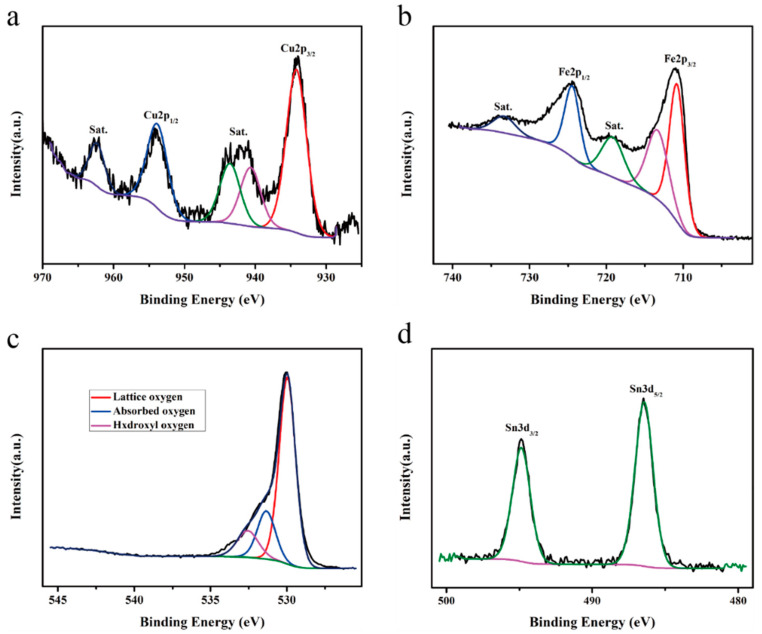
XPS spectra of the 2 mol% Sn^2+^-doped CuFe_2_O_4_ hollow microsphere sample: (**a**) Cu 2p spectrum, (**b**) Fe 2p spectrum, (**c**) O 1s spectrum, and (**d**) Sn 3d spectrum.

The XPS spectra of the CuFe_2_O_4_ hollow microsphere sample doped with 2 mol% Sn^2+^ are shown in Figure 11. In the deconvoluted Cu 2p spectrum (Figure 11a), the signals at 953.82 and 934.20 eV correspond to the Cu 2p_1/2_ and Cu 2p_3/2_ orbitals, respectively. The peaks at binding energies of 962.33, 943.57, and 940.66 eV represent vibrational satellite peaks that are consistent with previous literature reports and indicate that Cu still existed in the +2 valence state in the doped CuFe_2_O_4_ sample [26]. In the deconvoluted Fe 2p spectrum (Figure 11b), the peaks with binding energies of 713.33 and 710.81 eV can be attributed to Fe 2p_3/2_, the peak at 724.45 eV is assigned to Fe 2p_1/2_, and corresponding vibrational satellite peaks are present at 733.30 and 719.15 eV; these results indicate that Fe existed in the +3 valence state [27]. In the deconvoluted O 1s spectrum (Figure 11c), the three peaks at 532.56, 531.34, and 530.04 eV correspond to hydroxyl oxygen, surface-adsorbed oxygen, and lattice oxygen species, respectively [28], in proportions of 12.51%, 18.05%, and 69.44%, respectively, determined by measuring the corresponding XPS peak areas. Moreover, in the deconvoluted Sn 3d spectrum (Figure 11d), the peaks observed at 494.85 and 486.44 eV can be attributed to the Sn 3d_3/2_ and Sn 3d_5/2_ orbitals, respectively, and are consistent with previous literature, indicating that Sn was present in the +2 valence state [29]. According to relevant literature reports, the XPS binding energies of Sn 3d_3/2_ and Sn 3d_5/2_ orbitals of Sn^4+^ are 495.55 eV and 487.12 eV, respectively, which are not at the same position as the XPS binding energy of Sn^2+^ [30,31,32]. Thus, the XPS and EDS results (Figure 5) demonstrate the successful preparation of CuFe_2_O_4_ hollow microsphere samples doped with 2 mol% Sn^2+^.

### 3.2. Evaluation of the Gas-Sensing Performance

**Figure 12:**
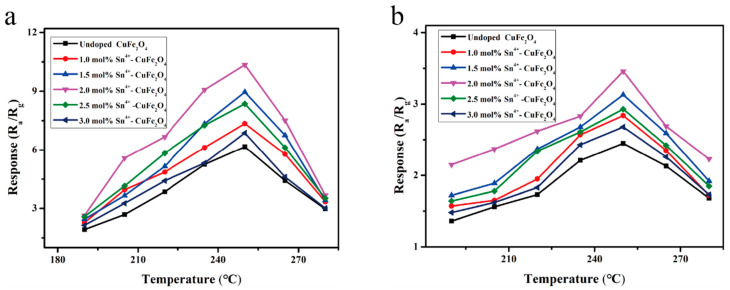
Response curves of the Sn^4+^-doped CuFe_2_O_4_ sensors to (**a**) 1 ppm DMMP and (**b**) 2-CEES under different operating temperatures.

Gas sensors were fabricated to detect DMMP (for sarin) and 2-CEES (for mustard gas) using the CuFe_2_O_4_ materials prepared using six different Sn^4+^ doping levels. The response curves of the six sensors to 1 ppm DMMP gas were recorded in the working temperature range of 190–280 °C (Figure 12a). All sensors exhibited an initially increasing then decreasing response trend as the operating temperature increased; the optimal operating temperature was 250 °C. In addition, the sensor performance initially increased, then decreased, as the Sn^4+^ doping level increased, with the highest response value to 1 ppm DMMP at 250 °C recorded for the 2 mol% Sn^4+^-doped sensor (i.e., 10.35); this value was ~1.7-fold higher than that for the undoped CuFe_2_O_4_ gas sensor (i.e., 6.16). Similarly, Figure 12b shows the response curves recorded for the six CuFe_2_O_4_-based gas sensors in the presence of 1 ppm 2-CEES gas under different temperature conditions. Similar to the DMMP responses, the sensor responses to 2-CEES varied with the working temperature and Sn^4+^ doping level. The highest response value (3.46) was obtained at 250 °C for the sensor containing 2 mol% Sn^4+^; this value was ~1.7-fold higher than that of the undoped sensor. These results clearly indicated that the response characteristics of the CuFe_2_O_4_ gas sensor were enhanced by Sn^4+^ doping. Notably, the response value obtained in the presence of 1 ppm DMMP (10.35) was nearly 3-fold greater than that obtained in the presence of 1 ppm 2-CEES (3.46); however, the sensor demonstrated suitable selectivity toward both agents.

**Figure 13:**
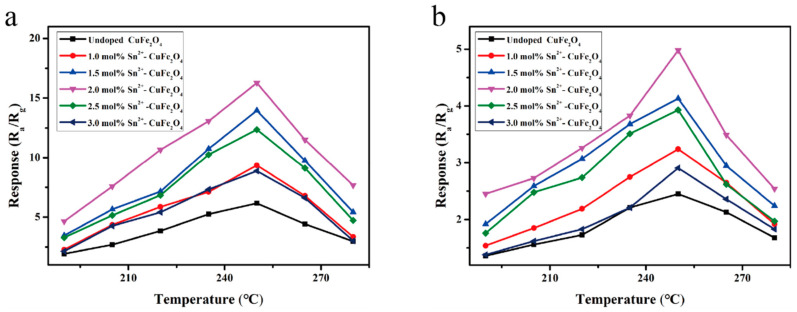
Response curves of the Sn^2+^-doped CuFe_2_O_4_ sensors to (**a**) 1 ppm DMMP and (**b**) 2-CEES under different operating temperatures.

Next, gas sensors were fabricated for the detection of DMMP and 2-CEES using the CuFe_2_O_4_ materials prepared using six different Sn^2+^ doping levels. The response curves of the six sensors to 1 ppm DMMP gas were recorded in the working temperature range of 190–280 °C (Figure 13a). All sensors exhibited an initially increasing then decreasing response trend as the operating temperature increased; the optimal operating temperature was 250 °C. In addition, the sensor performance initially increased, then decreased, as the Sn^2+^ doping level increased. The highest response value to 1 ppm DMMP at 250 °C was recorded for the 2 mol% Sn^2+^-doped sensor (i.e., 16.27); this value was ~2.6-fold higher than that for the undoped CuFe_2_O_4_ gas sensor (i.e., 6.16). Similarly, Figure 13b shows the response curves recorded for the six CuFe_2_O_4_-based gas sensors in the presence of 1 ppm 2-CEES gas under different temperatures. As in the case of DMMP, the sensor response varied with the working temperature and the Sn^2+^ doping level. The highest response value (4.98) was obtained at 250 °C for the sensor containing 2 mol% Sn^2+^; this value was ~2.6-fold higher than that of the undoped sensor and ~1.6-fold higher than that of the 2 mol% Sn^4+^-doped CuFe_2_O_4_ sensor, indicating a high response. These results clearly indicated that the response characteristics of the CuFe_2_O_4_ gas sensor were enhanced by Sn^2+^ doping. Notably, the response value obtained in the presence of 1 ppm DMMP (16.27) was nearly 3.3-fold greater than that obtained in the presence of 1 ppm 2-CEES (4.98); however, the sensor demonstrated suitable selectivity toward both agents.

**Figure 14:**
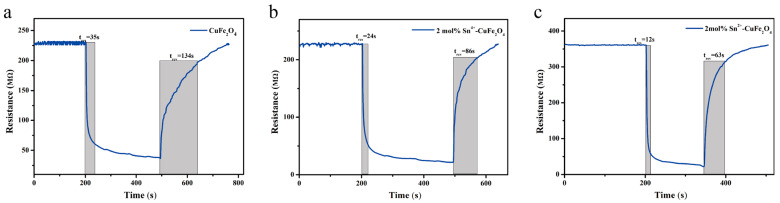
Response–recovery curves of (**a**) the undoped CuFe_2_O_4_ gas sensor, (**b**) the 2 mol% Sn^4+^-doped CuFe_2_O_4_ sensor, and (**c**) the 2 mol% Sn^2+^-doped CuFe_2_O_4_ sensor when exposed to 1 ppm DMMP gas at the optimal operating temperature of 250 °C.

The response–recovery curves of the undoped CuFe_2_O_4_ sensor, 2 mol% Sn^4+^-doped CuFe_2_O_4_ sensor, and 2 mol% Sn^2+^-doped CuFe_2_O_4_ sensor when exposed to 1 ppm DMMP gas at the optimal operating temperature of 250 °C are shown in Figure 14a, Figure 14b and Figure 14c, respectively. These results indicated response/recovery times of 35/134, 24/86, and 12/63 s for the undoped, Sn^4+^-doped, and Sn^2+^-doped sensors, respectively, as detailed in Table 3.

Considering the data presented in Figure 14 and Table 3, the response values and response–recovery characteristics of the 2 mol% Sn^2+^-doped CuFe_2_O_4_ were clearly superior to those of the undoped CuFe_2_O_4_ sensor. The obtained XPS results indicated that this superiority can be attributed to the relatively large surface oxygen adsorption ratio of the 2 mol% Sn^2+^-doped CuFe_2_O_4_, as shown in Table 4. This large ratio also accounted for the superior gas-sensing performance of the 2 mol% Sn^2+^-doped CuFe_2_O_4_ sensor compared to that of the 2 mol% Sn^4+^-doped sensor. Although lattice oxygen species cannot generally participate in gas reactions, an increase in the hydroxyl group content can promote the surface adsorption of gases and increase the number of reaction active sites. Indeed, surface-adsorbed oxygen species directly participate in gas oxidation/reduction reactions, with a higher proportion of surface-adsorbed oxygen species promoting gas reactions to alter the sensor resistance [33,34].

**Figure 15:**
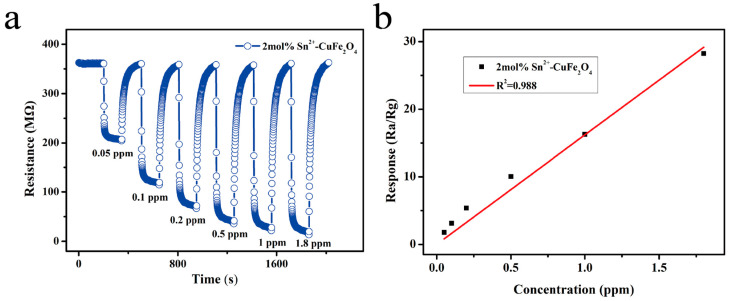
(**a**) Response–recovery curves and (**b**) linear fitting plot for the 2 mol% Sn^2+^-doped CuFe_2_O_4_ sensor during the detection of DMMP (0.05–1.8 ppm) at 250 °C.

The performance of the 2 mol% Sn^2+^-doped CuFe_2_O_4_ sensor was subsequently evaluated at the optimal operating temperature of 250 °C over a DMMP concentration range of 0.05–1.8 ppm. As shown in Figure 15a, the response of the sensor increased with the DMMP concentration; the corresponding response values are listed in Table 5. Notably, the response to 0.05 ppm DMMP (i.e., the IDLH value of sarin) was 1.76, indicating that the sensor limit of detection was <0.05 ppm for DMMP. As shown in Figure 15b, the sensor exhibited suitable linearity (*y* = 16.201*x*) over DMMP concentrations of 0.05–1.8 ppm, with a linear correlation coefficient of 0.988, indicating the practical potential of this sensor.

**Figure 16:**
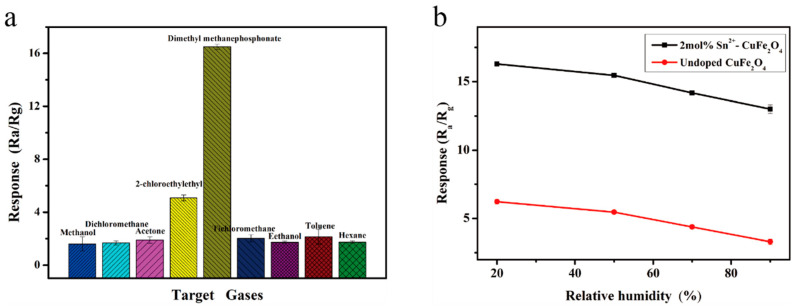
(**a**) Response of the 2 mol% Sn^2+^-doped CuFe_2_O_4_ sensor to different gases (1 ppm) at the optimal operating temperature of 250 °C. (**b**) Responses of the undoped and 2 mol% Sn^2+^-doped CuFe_2_O_4_ sensors to 1 ppm DMMP under different humidity conditions at 250 ° C.

The 2 mol% Sn^2+^-doped CuFe_2_O_4_ sensor was also used to detect 1 ppm of seven common volatile interfering gases (methanol, ethanol, acetone, dichloromethane, trichloromethane, toluene, and n-hexane), as well as 2-CEES or DMMP, at 250 °C. Figure 16a shows that the prepared sensor exhibited the highest response value of 16.27 to DMMP, followed by a response value of 4.98 to 2-CEES, and significantly lower response values to the other interfering gases (2.14, 2.02, 1.90, 1.82, 1.73, 1.68, and 161 for toluene, trichloromethane, acetone, ethanol, n-hexane, dichloromethane, and methanol, respectively), thereby demonstrating the selectivity of the 2 mol% Sn^2+^-doped CuFe_2_O_4_ sensor toward DMMP despite these interfering gases. As shown in Figure 16b, the detection of DMMP using the 2 mol% Sn^2+^-doped CuFe_2_O_4_ sensor was also evaluated under different relative humidity (RH) conditions of 20%, 50%, 70%, and 90%. The response value of the undoped CuFe_2_O_4_ sensor gradually decreased as the RH increased, reaching 3.32 at 90% RH, representing a ~50% reduction, whereas the response value of the 2 mol% Sn^2+^-doped CuFe_2_O_4_ sensor decreased by only ~20% from 16.27 to 13.03. These reductions with increasing RH were attributed to competitive adsorption between the target gas and water vapor molecules at the adsorbed oxygen sites on the sensor material [35]. Thus, the 2 mol% Sn^2+^-doped CuFe_2_O_4_ sensor was significantly less affected by the RH than the undoped sensor owing to the increased abundance of material defects introduced by Sn^2+^ doping. Water molecules are primarily adsorbed at these defect sites, thereby reducing the number of water molecules competing with the target gas at the oxygen sites and enhancing the humidity resistance of the sensor [36,37,38].

**Figure 17:**
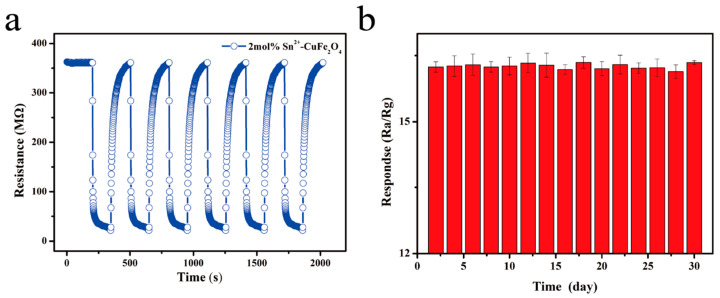
(**a**) Repeatability testing and (**b**) stability testing performed over 30 d for the detection of 1 ppm DMMP using the 2 mol% Sn^2+^-doped CuFe_2_O_4_ sensor at 250 °C.

Finally, a six-cycle response–recovery test was performed on the 2 mol% Sn^2+^-doped CuFe_2_O_4_ sensor in the presence of 1 ppm DMMP at 250 °C. As shown in Figure 17a, the response–recovery curve remained unchanged, indicating the repeatability of this sensor when detecting DMMP. Additionally, the response value of the sensor (Figure 17b) exhibited no significant fluctuations in response over 30 d. Critically, these results confirmed the stability of the sensor for the long-term stable monitoring of DMMP. A performance comparison of the 2 mol% Sn^2+^-doped CuFe_2_O_4_ sensor with the literature is shown in Table 6.

### 3.3. Mechanistic Analysis

**Figure 18:**
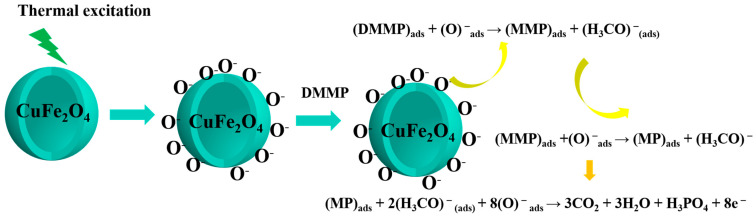
Gas-sensing mechanism diagram of the CuFe_2_O_4_ hollow microspheres in the presence of DMMP.

The DMMP system was taken as an example to analyze the sensing mechanism underlying the CuFe_2_O_4_-based gas sensor. As shown in Figure 18, the gas-sensing mechanism was attributed to a change in resistance on the CuFe_2_O_4_ material surface owing to the adsorption of DMMP on its surface oxygen species.

Specifically, in an air atmosphere, oxygen molecules adsorb on the surface of CuFe_2_O_4_ and take electrons from its conduction band, producing more actively adsorbed oxygen species (i.e., O_2_^−^, O^−^, and O^2−^) [39,40]. This generates an electron-depletion layer on the surface of the material that increases the material resistance. According to previous studies, the adsorbed oxygen species primarily exist in the form of O_2_^−^ at temperatures below 150 °C, whereas O^−^ dominates at temperatures between 150 and 400 °C, and O^2−^ is the most abundant species at temperatures above 400 °C [41]. These reaction processes are shown in Equations (1)–(4):

(1) $$O_{2}(gas)\to O_{2}(ads)$$

(2) $$O_{2}(ads)+e^{-}\to O_{2}^{-}(ads)$$

(3) $$O_{2}^{-}(ads)+e^{-}\to 2O^{-}(ads)$$

(4) $$O^{-}(ads)+e^{-}\to O^{2-}(ads)$$

With these reactions in mind, the surface decomposition of DMMP on CuFe_2_O_4_ was considered to proceed with the release of methanol in each step as follows: DMMP → MMP → MP, where MMP is methyl methylphosphonate and MP is methylphosphonate. In the first step, DMMP adsorbs onto the surface of CuFe_2_O_4_, where it undergoes partial oxidation upon reaction with the active oxygen species. This reaction generates MMP along with methanol, and the subsequent step proceeds in a similar manner to generate MP, followed by H_3_PO_4_ and CO_2_. Each step releases electrons back into the conduction band of the sensor material, thereby reducing the thickness of the electron-depletion layer and decreasing the material resistance [42]. These reactions are summarized in Equations (5)–(7):

(5) $$DMMP+O^{-}\to MMP+H_{3}CO^{-}$$

(6) $$MMP+O^{-}\to MP+H_{3}CO^{-}$$

(7) $$MP+2(H_{3}CO^{-})+8O^{-}\to 3CO_{2}+3H_{2}O+H_{2}O+H_{3}PO_{4}+8e^{-}$$

Consequently, the enhanced sensor performance and response observed after Sn^2+^ doping can be attributed to the generation of abundant surface-adsorbed oxygen species, which react with DMMP and promote charge transfer on the surface. In addition, Sn^2+^ can act as an electron donor and a Lewis basic site on the sensor surface, further promoting the adsorption of DMMP and its intermediate breakdown products. As a result, greater quantities of adsorbed oxygen species are consumed, resulting in enhanced electron transfer and ultimately increasing the sensor response.

## 4. Conclusions

This study developed a (sarin) gas sensor by preparing graded CuFe_2_O_4_ hollow microspheres via a solvothermal approach, then doping them with different ratios of Sn^4+^ or Sn^2+^. The prepared materials were first characterized to confirm successful doping, then the corresponding gas sensors were fabricated for testing. In tests using DMMP as a substitute for the highly toxic sarin nerve agent, the CuFe_2_O_4-_based sensor doped with 2 mol% Sn^2+^ exhibited excellent gas-sensing performance in response to a 1 ppm concentration of DMMP with response and recovery times of 12 and 63 s, respectively. Notably, its response to 1 ppm DMMP (16.27) was 3.3-fold higher than that to 1 ppm 2-CEES (4.98). Additionally, the sensor response to DMMP exhibited suitable linearity for concentrations between 0.05 and 1.8 ppm, and the sensitivity of the response to 0.05 ppm DMMP (i.e., the IDLH value of sarin) was 1.76, indicating that the DMMP detection limit was <0.05 ppm. Furthermore, the 2 mol% Sn^2+^-doped sensor exhibited superior response–recovery characteristics and enhanced moisture resistance compared to the undoped sensor owing to the higher proportion of surface-adsorbed oxygen species on the former, which directly participated in chemical reactions with DMMP. Moreover, Sn^2+^ provided electrons and acted as a Lewis basic site on the sensor surface, further enhancing the adsorption of DMMP and its intermediate decomposition products. This increased consumption of adsorbed oxygen, thereby promoting electron transfer and enhancing the sensor response. These results are relevant to addressing the dangers associated with sarin gas through the development of appropriate technologies for detecting this nerve agent.

## Figures and Tables

**Table 1:** EDS results obtained for the Sn^4+^-doped CuFe_2_O_4_ samples.

Material	Cu	Fe	O	Sn
Pure-CuFe_2_O_4_	13.87%	27.53%	58.60%	0
1 mol% Sn^4+^-CuFe_2_O_4_	13.60%	26.89%	59.40%	0.11%
1.5 mol% Sn^4+^-CuFe_2_O_4_	11.76%	28.91%	59.20%	0.13%
2 mol% Sn^4+^-CuFe_2_O_4_	12.72%	28.21%	58.90%	0.17%
3 mol% Sn^4+^-CuFe_2_O_4_	11.92%	29.52%	58.30%	0.26%
4 mol% Sn^4+^-CuFe_2_O_4_	12.15%	29.32%	58.20%	0.33%

**Table 2:** EDS results obtained for the Sn^2+^-doped CuFe_2_O_4_ samples.

Material	Cu	Fe	O	Sn
Pure CuFe_2_O_4_	13.70%	27.80%	58.50%	0
1 mol% Sn^2+^-CuFe_2_O_4_	12.74%	32.80%	54.38%	0.08%
1.5 mol% Sn^2+^-CuFe_2_O_4_	13.16%	28.70%	58.02%	0.12%
2 mol% Sn^2+^-CuFe_2_O_4_	12.10%	29.60%	58.12%	0.18%
3 mol% Sn^2+^-CuFe_2_O_4_	13.20%	31.50%	55.05%	0.25%
4 mol% Sn^2+^-CuFe_2_O_4_	12.90%	29.20%	57.56%	0.34%

**Table 3:** Gas-sensing properties of the undoped, 2 mol% Sn^4+^-doped, and 2 mol% Sn^2+^-doped CuFe_2_O_4_ sensors when exposed to 1 ppm DMMP gas at 250 °C.

Appearance	Response Value	Response/Recovery Time
Undoped CuFe_2_O_4_	6.16	35 s/134 s
CuFe_2_O_4_ doped with 2 mol% Sn^4+^	10.35	24 s/86 s
CuFe_2_O_4_ doped with 2 mol% Sn^2+^	16.27	12 s/63 s

**Table 4:** Comparison of the oxygen species ratios in the undoped, 2 mol% Sn^4+^-doped, and 2 mol% Sn^2+^-doped CuFe_2_O_4_ materials.

Appearance	Lattice Oxygen	Surface Adsorbed Oxygen	Hydroxyl Oxygen
Undoped CuFe_2_O_4_	79.34%	11.23%	9.43%
CuFe_2_O_4_ doped with 2 mol% Sn^4+^	73.49%	15.37%	11.14%
CuFe_2_O_4_ doped with 2 mol% Sn^2+^	69.44%	18.85%	12.51%

**Table 5:** Response values of the 2 mol% Sn^2+^-doped CuFe_2_O_4_ sensor during the detection of DMMP (0.05–1.8 ppm) at 250 °C.

DMMP Concentration	0.05 ppm	0.1 ppm	0.2 ppm	0.5 ppm	1 ppm	1.8 ppm
Response value	1.76	3.12	5.37	10.03	16.27	28.24

**Table 6:** Performance comparison of the 2 mol% Sn^2+^-doped CuFe_2_O_4_ sensor with the literature.

Material	Operating Temperature	Concentration	Response Value	Limit of Detection	Literature
SnO_2_	350 °C	0.5 ppm	0.4	0.2 ppm	[2]
4% ZnO-SnO_2_	300 °C	1 ppm	0.55	0.1 ppm	[4]
2 mol% Sn^2+^-CuFe_2_O_4_	250 °C	1 ppm	16.27	0.05 ppm	This paper

## Data Availability

Data are contained within the article.
